# A rare sporadic pancreatic desmoid fibromatosis with splenic vein invasion diagnosed by CT scan-guided core needle biopsy: a case report with possible differential diagnosis from metastatic colorectal or renal cancer

**DOI:** 10.1093/jscr/rjab257

**Published:** 2021-06-26

**Authors:** Alberto Meyer, Paulo Szajnbok, Andreas Johann Molnar Koszka, Daniela Pezzutti, Vanderlei Segatelli, José Monteiro

**Affiliations:** Departamento de Gastroenterologia, Hospital das Clínicas, HCFMUSP, São Paulo, Brazil; Division of Digestive Surgery, Samaritano Hospital, São Paulo, Brazil; Hospital Israelita Albert Einstein, São Paulo, Brazil; Division of Digestive Surgery, Samaritano Hospital, São Paulo, Brazil; Americas Oncologia, Samaritano Hospital, São Paulo, Brazil; Pathology Department, Hospital Israelita Albert Einstein, São Paulo, Brazil; Hospital Israelita Albert Einstein, São Paulo, Brazil; Samaritano Hospital, São Paulo, Brazil

## Abstract

Desmoid tumor is a rare fibroblastic proliferation with a variable and often unpredictable clinical course that arises in the deep soft tissues and is characterized by infiltrative growth with tendency to local recurrence but not to metastasize. A 49-year-old man was referred for a second opinion regarding a pancreatic mass. With a personal neoplastic background of two different tumors, we considered as a high probability of being metastatic of his previous colorectal or renal cancers, in a peritoneal implant. Due to the unclear origin and nature of the mass, we opted for requesting a computed tomography (CT)-guided core needle biopsy that could eventually lead to a surgical and/or chemotherapy treatment. So far, this is the first case of pancreatic desmoid fibromatosis with splenic vein invasion diagnosed by CT scan-guided core needle biopsy. Surgery should be performed by an experienced surgeon as first-line therapy, provided expected surgical morbidity is limited.

## INTRODUCTION

Desmoid tumor (DT) is a rare monoclonal, fibroblastic proliferation with a variable and often unpredictable clinical course [1]. About 5–10% of DT may occur in association to familial adenomatous polyposis (FAP), but they present typically as sporadic cases [2, 3]. Therefore some authors recommend the research for FAP in patients with DT [4].

Desmoids in FAP arise from adenomatous polyposis coli (APC) inactivation and subsequent accumulation of beta-catenin in cells [3, 5]. In contrast, APC mutations are uncommon in sporadic desmoids, which usually arise from mutations in the gene for beta-catenin (CTNNB1), and positive nuclear staining for beta-catenin is reported in about 80–100% of cases [3, 5].

Cross-sectional imaging of the affected area with computed tomography (CT) or magnetic resonance imaging is needed to evaluate the involvement of the tumor, in order to assess resectability. There are no imaging patterns that clearly differentiate between DT and malignant soft tissue tumors.

The diagnosis of a DT can only be established by histological findings of a biopsy specimen. However, for experienced pathologists, even tissue from a core biopsy may be sufficient to the correct diagnosis.

DT involving the pancreas is very rare with only few cases previously reported in the English-language literature [6], and the splenic vein invasion is an uncommon finding [7].

In this case, a CT scan-guided core needle biopsy gave an adequate cellular sample allowing accurate diagnosis by immunohistochemistry (IHC) staining and molecular testing.

## CASE REPORT

A 49-year-old man was referred for a second opinion regarding a pancreatic mass. The patient did not complain of abdominal pain, nausea, vomiting, chills, fever, bloating, diarrhea, anorexia, fatigue, hematochezia and cutaneous itching. There was no history of diabetes, without reference to using drugs, alcohol or tobacco.

In 2019 the patient had the diagnosis of an ascendent colon cancer and underwent a laparoscopic right colectomy with an extended colon resection performed until close to the splenic angle. Histopathology showed a well-differentiated adenocarcinoma of the colon with the 73 resected lymph nodes (LNs) negative for metastasis, Stage IIA. No evidence of Gardner’s syndrome or FAP was found.

Genetic screening did not show any pathogenic variants but identified one of uncertain significance in BMPR1A gene, which is associated with autosomal dominant juvenile polyposis syndrome.

At the end of 2020, the patient had the diagnosis of a right renal tumor and underwent a robotic partial right nephrectomy in a zero-ischemia procedure. The pathologic results were a renal cell carcinoma (RCC) of clear cells, Stage I, Fuhrman grade II.

The complete blood count, complete metabolic and coagulation panels, enzyme and tumor markers (CA l9-9, CA 50, CA 24-2, CEA) were all normal.

**Figure 1:**
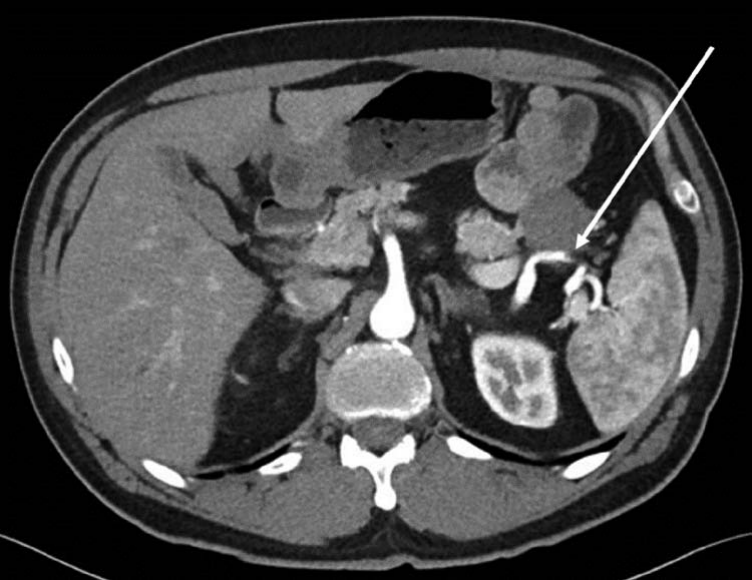
Axial CT scan of abdomen, selected image from a pancreatic protocol. Early arterial phase during which there is opacification of the arterial structure (arrow).

**Figure 2:**
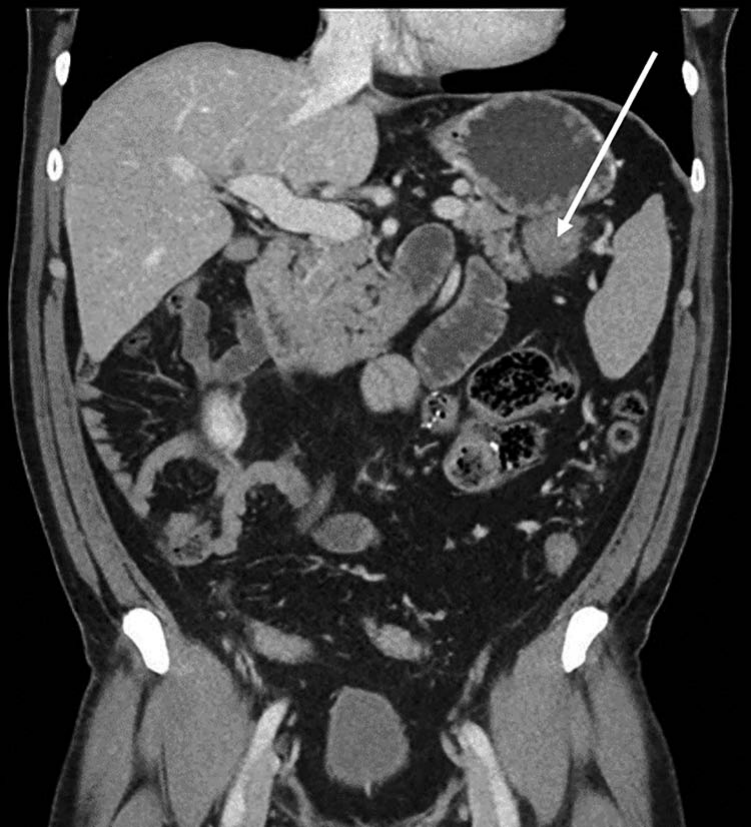
Coronal CT scan of abdomen, selected image from a pancreatic protocol, portal venous phase. A 3.7-cm mass, between the tail of the pancreas and the splenic hilum, corresponding to a peritoneal implant (arrow).

**Figure 3:**
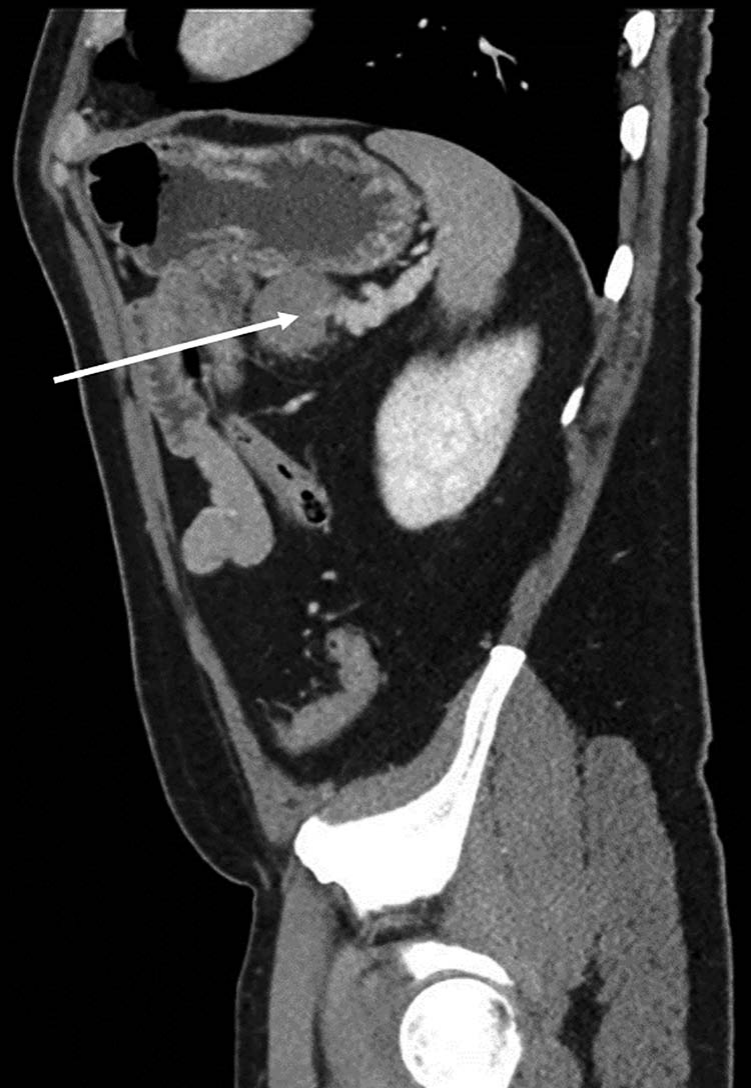
Sagital CT scan of abdomen, selected image from a pancreatic protocol, late arterial/pancreatic phase. A 3.7-cm mass, between the tail of the pancreas and the splenic hilum, causing a narrowing of the splenic vein and splenomegaly (arrow).

Abdominopelvic CT scan disclosed a 3.7-cm mass, between the tail of the pancreas and the splenic hilum, causing a narrowing of the splenic vein and splenomegaly (Figs 1–3).

With that personal neoplastic background of two different tumors, these findings were considered as a high probability of being metastatic colorectal or renal lesion, in a peritoneal implant. Due to the unclear origin and nature of the mass, we opted for requesting a CT-guided core needle biopsy that could eventually lead to a surgical and/or chemotherapy treatment.

**Figure 4:**
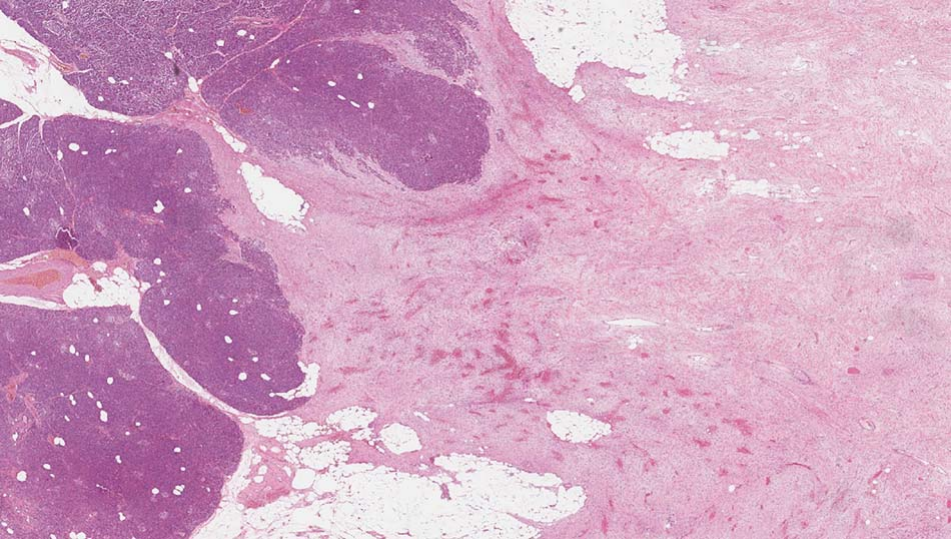
Microscopic study showing fusocellular proliferation with collagen-rich stroma, infiltrative in adipose tissue and pancreas (hematoxylin–eosin [HE]).

**Figure 5:**
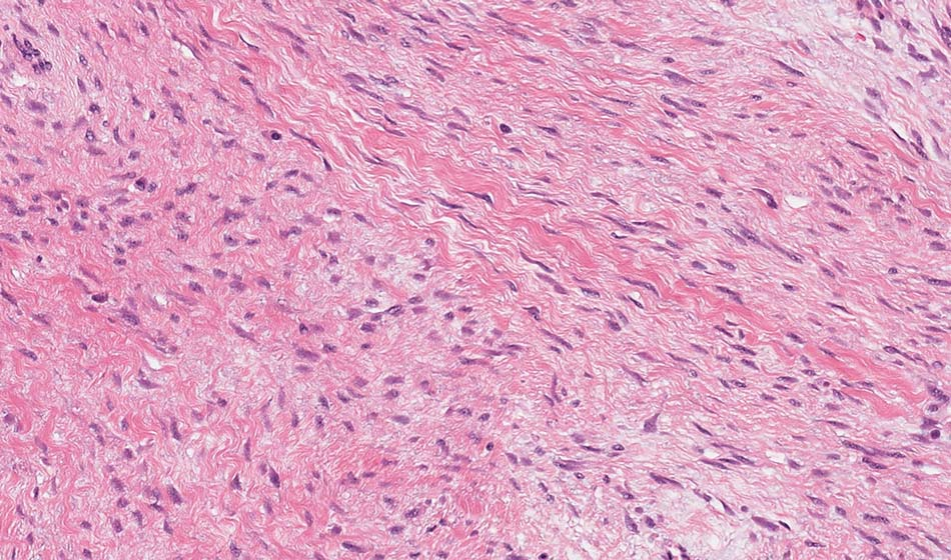
Microscopic study showing fusocellular proliferation with collagen-rich stroma, infiltrative in adipose tissue and pancreas (HE).

**Figure 6:**
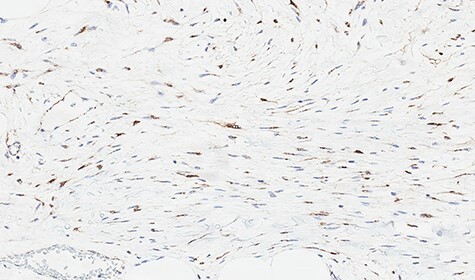
Immunohistochemical study showing positive nuclear expression for beta-catenin.

Microscopically, the lesion presented as proliferation of spindle cells without atypia, a low mitotic index and a fibrous stroma rich in collagen (Figs 4 and 5). IHC specimen showed positive nuclear immunoexpression for beta-catenin and focal immunoexpression for smooth muscle actin (Fig. 6). The immunoexpressions for cytokeratins, desmin, caldesmon, protein S CD117, DOG1, CD34, STAT6 and S100 were all negative.

**Figure 7:**
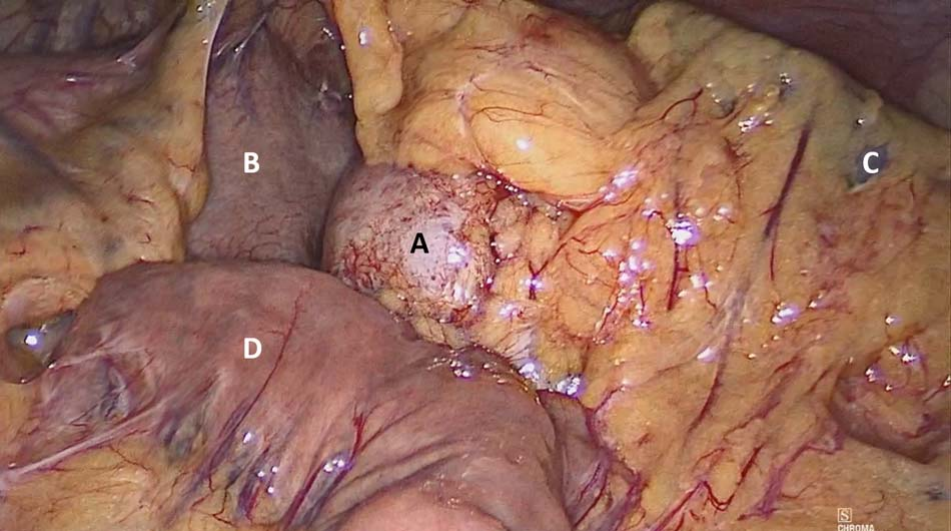
Laparoscopic view of (**A**) nodular lesion, (**B**) stomach, (**C**) spleen and (**D**) small intestine.

**Figure 8:**
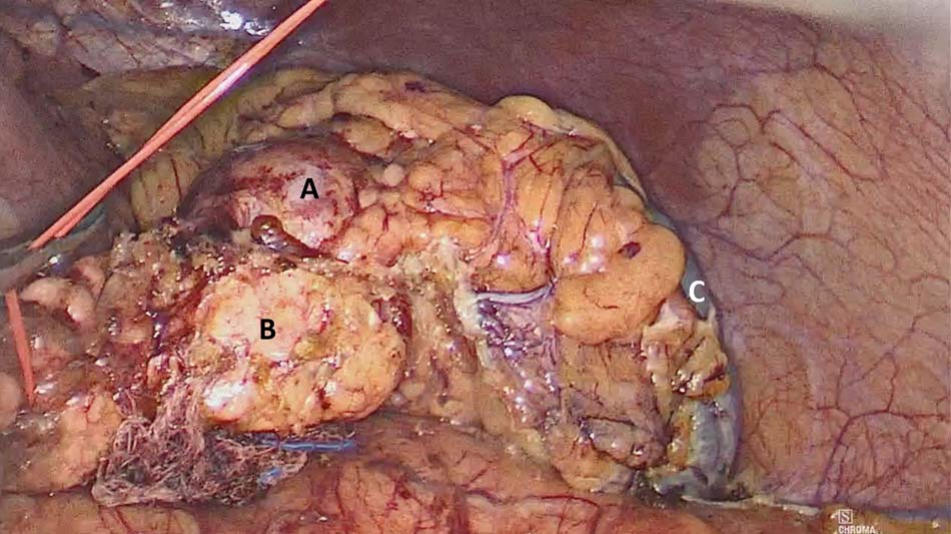
Laparoscopic view of (**A**) nodular lesion, (**B**) vessel loop encircling the pancreas and (**C**) spleen.

The patient underwent laparoscopic distal pancreatectomy with splenectomy (Figs 7 and 8) with the same surgical team. The patient had an uneventful postoperative recovery and was discharged within 48 hours.

**Figure 9:**
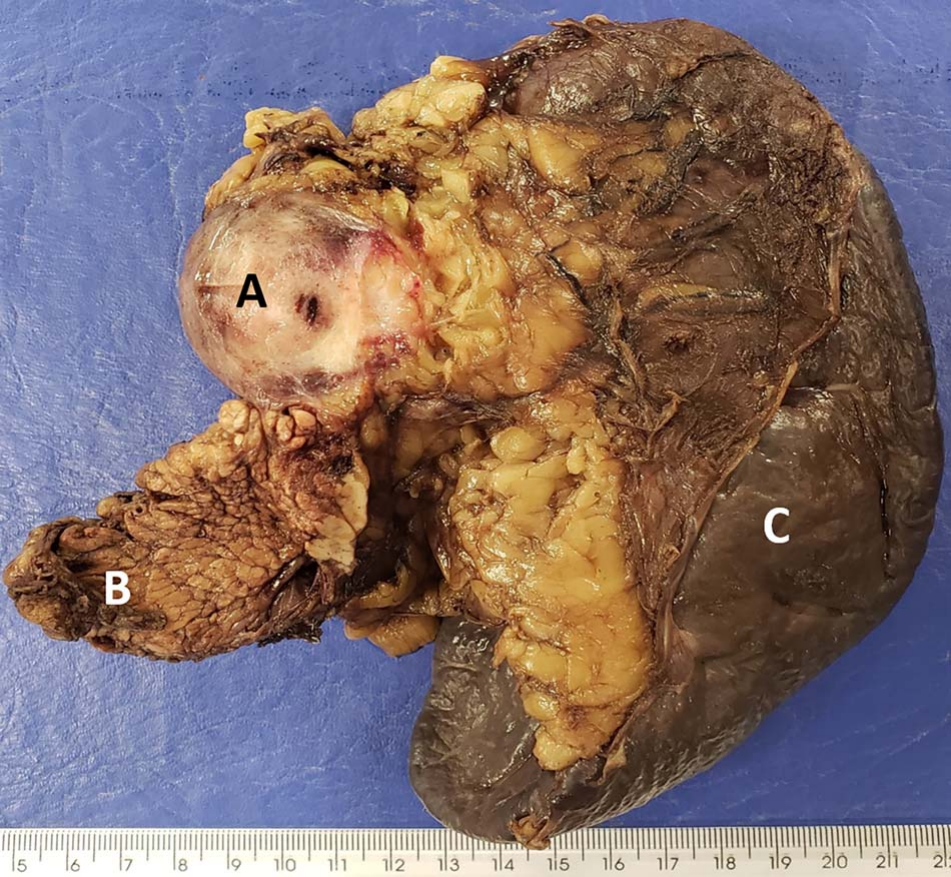
Macroscopic image showing firm and brown (**A**) nodular area, on the periphery of the (**B**) caudal region of the pancreas and (**C**) spleen.

The macroscopic anatomopathological study showed a 4.3 cm diameter nodular lesion, consisting of a firm and brownish tissue, ill-defined fibrous mass in the pancreas that was diffusely infiltrative on gross and microscopic examination (Fig. 9). Histologic examination and IHC were consistent with DT. There was no involvement of the surgical margins, spleen or mesenteric LNs.

## DISCUSSION

DTs (also called aggressive fibromatosis) are benign, slow-growing fibroblastic neoplasms with no metastatic potential but a tendency for local recurrence (8–31%) [8], even after complete surgical excision. Even though histologically benign, they are locally infiltrative and can cause death through invasion of adjacent vital structures and organs [9].

Fine needle aspiration (FNA) or core needle biopsy can be an important tool for preoperative diagnosis of intra-abdominal DT. Previous studies of DT in FNA specimens are relatively rare but do offer some guidance regarding helpful features and frequent pitfalls [6]. Obtaining adequate specimens to allow IHC staining was also more likely to give a definitive preoperative diagnosis and has a clear role in distinguishing DT from other spindle cell tumors [10].

In addition, colorectal cancer (CRC) is a leading cause of cancer-related deaths worldwide, and its incidence has been increasing [11]. Peritoneal dissemination is present in 4–10% of all CRCs, most of which are T3 or T4 CRCs [12]. Approximately one-fifth of initially M0 (TNM Classification of Malignant Tumors, seventh edition) CRC patients who developed metastases during follow-up is diagnosed with peritoneal carcinomatosis. Prognosis of these patients is poor with a median survival of 6 months after diagnosis. Early diagnosis and identification of patients at high risk of developing metachronous peritoneal carcinomatosis is important, as selected patients may benefit from potentially curative treatments such as cytoreductive surgery combined with hyperthermic intraperitoneal chemotherapy [13].

Regarding kidney cancers, a study [14] that included 1147 patients who underwent unilateral RCC nephrectomies (T1–T2, N0, and M0) concluded that age over 45 years is an independent predictor of survival and is associated with a higher mortality incidence in localized RCC.

Therefore, DT involving the pancreas, because of its rarity and often nonspecific presentation, represents a distinct diagnostic challenge [3, 5]. So far, this is the first case of pancreatic desmoid fibromatosis with splenic vein invasion diagnosed by CT scan-guided core needle biopsy.

In summary, when interventional treatment for DT is required, surgery should be performed by an experienced surgeon as first-line therapy, since undesirable consequences are unexpected.

## AUTHORS' CONTRIBUTIONS

All the authors testified to the care of the patient and the writing of the manuscript. The authors have read and agreed with the contents of the manuscript.

## Conflict of Interest Statement

None declared.

## CONSENT

Written informed consent was obtained from the patient for publication of this case report and any accompanying images. A copy of the written consent is available for review by the Editor-in-Chief of this journal on request.
